# The importance of heteroresistance and efflux pumps in bedaquiline-resistant *Mycobacterium tuberculosis* isolates from Iran

**DOI:** 10.1186/s12941-024-00694-3

**Published:** 2024-04-25

**Authors:** Nahid Madadi-Goli, Kamal Ahmadi, Mansour Kargarpour Kamakoli, Mohsen Azizi, Sharareh Khanipour, Shahin Pourazar Dizaji, Mahshid Nasehi, Seyed Davar Siadat, Abolfazl Fateh, Farzam Vaziri

**Affiliations:** 1 Department of Mycobacteriology and Pulmonary Research, Pasteur Institute of Iran, No. 358, 12th Farvardin Ave., Jomhoori St, Tehran, 1316943551 Iran; 2 Microbiology Research Center (MRC), Pasteur Institute of Iran, Tehran, Iran; 3 Department of Microbiology, School of Medicine, Kermanshah University of Medical Sciences, Kermanshah, Iran; 4 Student Research Committee, Pasteur Institute of Iran, Tehran, Iran; 5 Department of Epidemiology and Biostatistics, School of Public Health, Iran University of Medical Sciences, Tehran, Iran; 6 Center for Communicable Diseases Control, Ministry of Health and Medical Education, Tehran, Iran

**Keywords:** *M. Tuberculosis*, Bedaquiline, Mixed infection, Heteroresistance, Efflux pump

## Abstract

**Background:**

Tuberculosis (TB) continues to pose a threat to communities worldwide and remains a significant public health issue in several countries. We assessed the role of heteroresistance and efflux pumps in bedaquiline (BDQ)-resistant *Mycobacterium tuberculosis* isolates.

**Methods:**

Nineteen clinical isolates were included in the study, of which fifteen isolates were classified as MDR or XDR, while four isolates were fully susceptible. To evaluate BDQ heteroresistance, the Microplate Alamar Blue Assay (MABA) method was employed. For screening mixed infections, MIRU-VNTR was performed on clinical isolates. Mutations in the *atpE* and *Rv0678* genes were determined based on next-generation sequencing data. Additionally, real-time PCR was applied to assess the expression of efflux pump genes in the absence and presence of verapamil (VP).

**Results:**

All 15 drug-resistant isolates displayed resistance to BDQ. Among the 19 total isolates, 21.05% (4/19) exhibited a heteroresistance pattern to BDQ. None of the isolates carried a mutation of the *atpE* and *Rv0678* genes associated with BDQ resistance. Regarding the MIRU-VNTR analysis, most isolates (94.73%) showed the Beijing genotype. Fifteen (78.9%) isolates showed a significant reduction in BDQ MIC after VP treatment. The efflux pump genes of *Rv0676c*, *Rv1258c*, *Rv1410c*, *Rv1634*, *Rv1819*, *Rv2459*, *Rv2846*, and *Rv3065* were overexpressed in the presence of BDQ.

**Conclusions:**

Our results clearly demonstrated the crucial role of heteroresistance and efflux pumps in BDQ resistance. Additionally, we established a direct link between the *Rv0676c* gene and BDQ resistance. The inclusion of VP significantly reduced the MIC of BDQ in both drug-susceptible and drug-resistant clinical isolates.

## Introduction

Tuberculosis (TB) is one of the main ten main global causes of mortality, remaining a significant public health concern in numerous countries [1]. The rise in drug-resistant TB, particularly pre-extensively drug-resistant TB (pre-XDR-TB), multidrug-resistant (MDR-TB), and XDR-TB, poses a critical threat to global TB control. MDR-TB is resistant against at least two key first-line drugs (isoniazid and rifampicin), pre-XDR-TB is resistant to both these drugs as well as fluoroquinolones, and XDR-TB is MDR-TB which exhibit resistance against any fluoroquinolone as well as at least one additional Group A drug (moxifloxacin, levofloxacin, linezolid and bedaquiline) [2].

Bedaquiline (BDQ), an oral diarylquinoline drug, has been approved by the Food and Drug Administration (FDA) to treat drug-resistant TB, the first new drug in after 40 years. It exhibits bactericidal anti-TB activity by targeting mycobacterial ATP synthase [3]. Resistance to BDQ is associated with specific genes, such as the *atpE* gene encoding F1/F0-ATPase synthase, which accounts for 30% of BDQ-resistant clinical isolates due to mutations in the C subunit of the enzyme. Another resistance gene, *Rv0678*, regulates the *MmpS5-MmpL5* efflux pump’s expression [4].

Infections with mixed strains of *Mycobacterium tuberculosis* can lead to false-negative results in drug resistance testing, affecting drug susceptibility testing (DST) accuracy [5]. Mixed infections can also promote the spread of drug-resistant strains and increase treatment failure rates [6]. Epidemiological and phylogenetic studies have reported an accepted MIRU-VNTR genotyping system with 24 and 15 locus sets, enabling the identification of mixed infections based on Variable Numbers of Tandem Repeats (VNTRs) of Mycobacterial Interspersed Repetitive Units (MIRUs) [7]. The 24-locus MIRU-VNTR is the gold standard for *M. tuberculosis* phylogenetic analysis [8].

Heteroresistant strains of *M. tuberculosis* consist of a mixture of susceptible and resistant subpopulations in clinical samples. Heteroresistance is considered a preliminary stage of total resistance, allowing bacteria to exploit growth opportunities even in the presence of antibiotics [9]. Efflux pumps in *M. tuberculosis* include major facilitator superfamily (MFS), proteobacterial antimicrobial compound efflux (PACE), multi-drug and toxic compound extrusion (MATE), Small Multidrug Resistance (SMR), ATP-binding cassette (ABC), and resistance-nodulation-division (RND) types [10].

We investigated the genotypic and phenotypic mechanisms of BDQ drug resistance in drug-resistant strains isolated from patients referred to the Mycobacteriology and Pulmonary Research Department, Pasteur Institute, Iran, between 2015 and 2021. The investigation involved determining the phenotypic and genotypic antibiotic resistance of MDR strains, assessing the effect of polyclonal infections, presence of heteroresistance to BDQ, and evaluating the role of efflux pumps as a resistance mechanism against BDQ.

## Methods

### Bacterial strains

In this study, a total of 19 clinical isolates were employed, out of which 15 were classified as MDR, or XDR, while the remaining four isolates were fully susceptible. The isolates examined in this study were isolated from patients who had no history of using BDQ. Throughout the tests, the H37Rv (ATCC 27294) strain served as the reference strain. All of these isolates were collected between 2015 and 2021 from the Department of Mycobacteriology and Pulmonary Research, Pasteur Institute, Iran. To ensure ethical compliance, the Pasteur Institute Ethics Committee conducted thorough reviews of the study (Ethics approval number: IR.PII.REC.1399.055). Moreover, participants signed written informed consent.

### Detection of Bedaquiline Heteroresistance

At first, each of the samples was cultured in the Lowenstein-Johnson medium, then after the growth, 3 distinct colonies from each sample were subcultured individually in the Lowenstein-Johnson medium to expand further growth. BDQ was purchased from Sigma-Aldrich (St. Louis, MO, USA). The objective of this subculturing process was to assess BDQ resistance using the Microplate Alamar Blue Assay (MABA) method. Alamar Blue was purchased from AbD Serotec (Oxford, UK).

### DNA extraction and genotyping

At first, the colonies grown on the LJ medium were transferred into a microcentrifuge tube and DNA was extracted by the cetyltrimethylammonium bromide (CTAB) method [11]. For screening of mixed infection, MIRU-VNTR was done on isolates, revealing the polyclonal infection. Regarding MIRU-VNTRs, amplification of all 24 loci was done using the corresponding primers [12]. For each locus, deionized water and *M. tuberculosis* H37Rv DNA were added respectively as negative and positive controls. Separation of amplicons was done electrophoretically on 1.8% agarose gels, by a 100-bp DNA ladder as a size marker. Mixed infection was considered present when the specimen contained over one amplicon at two or more loci. All standard precautions were followed to avoid laboratory cross-contamination. The MIRU-VNTR results were analyzed by MIRU–VNTR plus [13].

### Sequencing of Rv0678 and atpE genes

After extracting the DNA of the isolates by the CTAB method, the mutation of the *atpE* and *Rv0678* genes was determined by next-generation sequencing as we described previously [14]. Briefly, libraries were loaded onto an Illumina NextSeq 500 instrument for a paired-end 2 × 151-bp run. The generated reads were mapped to the *M. tuberculosis* H37Rv genome (GenBank accession no. NC_000962.3) using the alignment software Burrow-Wheeler Aligner (BWA). Subsequently, mappings were refined with the toolkits Genome Analysis Toolkit (GATK), sambamba, and SAMtools.

### Determination of MICs and Verapamil (VP) effectiveness

The microplate Alamar Blue assay (MABA) was performed to determine the MICs for clinical isolates. Briefly, 100 µl of 7H9 medium was added to each well of 96-well polystyrene microtitre plates, except the peripheral wells, which were dispensed with 250 µl of sterilized water to prevent evaporation during incubation. Two-fold serial dilutions of BDQ (ranging from 64 to 0.0156 µg/ml) were prepared in the wells. The turbidity of each suspension was adjusted to a McFarland standard of 1.0 (3 × 10 ^8^ CFU/ml). 100 µl of *M. tuberculosis* inoculum was added to the wells. Each microplate contained a positive control well containing the bacterial suspension and a negative control well containing each antibiotic and culture medium. The plates were sealed and covered with a plastic bag and incubated at 37 °C for 7 days. After 7 days 50 µl of a mixture of 0.02% resazurin sodium salt (Sigma, USA) solution and Tween 80 were added to each well and again incubated overnight at 37 °C. A color change from blue to pink was considered as positive growth and the MIC was defined as the lowest drug concentration that prevented the color change. Each drug concentration was tested in triplicate, and all assays were performed twice to ensure accuracy. Isolates with BDQ MICs < 0.25 µg/mL were classified as vulnerable against BDQ [15]. The impact of VP as an efflux pump inhibitor (EPI), on the MIC of BDQ was investigated in this study [16]. VP was obtained from Sigma-Aldrich (St.Louis, MO, USA). For this purpose, the wells were filled with two-fold serial dilutions of BDQ both in the presence and absence of 64 µg/mL of VP. A previous study had already established 64 µg/mL as the optimal concentration for verapamil [17].

### Analysis of gene expression of drug efflux pumps

*M. tuberculosis* isolates underwent culturing in Middlebrook 7H9 broth (Sigma Aldrich, St. Louis, Mo., USA) with albumin-dextrose-catalase (ADC) supplement (Becton Dickinson, Oxford, UK), for the 15 MDR /XDR and 4 fully susceptible isolates for the total bacterial RNA extraction. These cultures were added with BDQ and BDQ + VP at sub-inhibitory levels (half of the MIC), followed by incubation at 37 °C for 7 days, and collection for RNA extraction. The TRIzol reagent (Life Technologies Corp., Carlsbad, USA) extracted the total bacterial RNA as per the manufacturer’s instructions. A nanophotometer assessed the integrity and quality of total RNA. Following treatment with DNase I (Invitrogen), RNA (1 µg) underwent cDNA synthesis as instructed (PrimeScript™ 1st strand cDNA Synthesis Kit, TAKARA). Quantitative reverse transcription PCR was done in a 20-µl system composed of 10 µl of 2X mixture, complementary DNA template (100 ng), SYBR Green, and 5 pmol of each primer set (Table 1) [18, 19]. The qPCR was performed on Rotor-Gene 6000 (Corbett Research Pty Ltd., Sydney, Australia). *SecA* was used as a housekeeping gene for normalization. The 2^−ΔΔCT^ method RNA calculated the relative quantification of gene expression in BDQ-induced strains in relation to the non-induced strain. In comparison with the non-induced control, an expression equal to one showed the same expression levels, an expression equal to or 4-fold increase, and an expression equal to or 4-fold decrease [20].

**Table 1:** Primers used in Real-Time PCR for the EP gene regulation study in Mtb

Gene	Sequence (5‘–3‘)	Amplicon size (bp)
*(Mmpl5) Rv0676c*	ATCCGCAGCTACTTCTACTGG TGCGCCTTCATGCTCTTC	212
*Rv3065*	AACCAGCCTGCTCAAAAG CAACCACCTTCATCACAGA	221
*Rv1258c*	AGTTATAGATCGGCTGGATG GTGCTGTTCCCGAAATAC	268
*Rv2459*	CATCTTCATGGTGTTCGTG CGGTAGCACACAGACAATAG	232
*Rv1218c*	CCGCAAGGCGTCTAGTGAA TGGACCCGTTGATGGAAAA	173
*Rv2846*	ATGGTAATGCCTGACATCC CTACGGGAAACCAACAAAG	131
*Rv1819c*	CGGTGATTTCTTTCACAGC CCGACAGATTCCATCCATT	351
*Rv1410c*	AGTGGGAAATAAGCCAGTAA TGGTTGATGTCGAGCTGT	198
*secA*	AGAGGTGTTCACGCCACTTACG GCTGGAGGCACTACTCAAGGAC	146

### Statistical analysis

The allelic table by Supply et al. was used to determine the number of repeats at each locus and clustering assessment was performed by the online tool at http://www.MIRU-VNTRplus.org [12]. SPSS 23 was used for data analysis. Descriptive analysis was performed by percentage, frequency, mean, figures, tables, etc. The Hunter Gaston discriminatory index illustrated the discriminatory power of 24 loci MIRU-VNTR [21].

The 2^−ΔΔCT^ method was applied to calculate the relative quantification of target gene regulation. Data analysis and drawing figures were performed through one-way ANOVA tests by GraphPad Prism 7. A p-value < 0.05 was regarded as significant.

## Results

### Despite the absence of mixed infection, there was a notable presence of high heteroresistance in the BDQ-resistant isolates

The study included a total of 19 selected isolates, comprising 4 fully susceptible isolates and 15 isolates with varying degrees of drug resistance (MDR/XDR). The proportional method, using Lowenstein-Jensen (LJ) medium supplemented with Isoniazid (INH) at 0.2 mg/liter, Rifampicin (RIF) at 40 mg/liter, Ethambutol (EMB) at 2 mg/liter, Kanamycin (KAN) at 20 mg/liter, Ofloxacin (OFX) at 4 mg/liter, Capreomycin (CAP) at 20 mg/liter, and Streptomycin (STR) at 4 mg/liter. Resistance was determined by bacterial growth equal to or exceeding 1% compared to growth in a drug-free Löwenstein-Jensen (LJ) medium used as a negative control for background growth. Based on the proportional antibiogram test results, all 15 drug-resistant isolates displayed resistance to INH, RIF, EMB, and BDQ. The resistance levels to KAN, OFX, CAP, and STR were found to be 46.6%, 53.3%, 80%, and 86.6%, respectively. Among the 4 fully susceptible isolates, 2 isolates (50%) were sensitive to BDQ. The fully susceptible isolates demonstrated sensitivity to all first- and second-line drugs tested. The MABA method was utilized to determine the MIC of BDQ for each strain on its three separate colonies. Among the 15 resistant isolates and 4 fully susceptible isolates, 21.05% (4/19) exhibited a heteroresistance pattern to BDQ, as shown in Table 2. Notably, none of the pan-susceptible or MDR/XDR isolates carried a mutation of the *atpE* and *Rv0678* genes associated with BDQ resistance.

**Table 2:** BDQ susceptibility profile, Heteroresistance, MIC findings and genotype in the studied isolates

Sample ID	BDQ	BDQ Heteroresistance	MICBDQ (µg/mL)	Genotype	Sample ID	BDQ	BDQ Heteroresistance	MIC BDQ (µg/mL)	Genotype
20	R	-	8	Beijing	33	R	*	0.5	Beijing
20 − 1	R		8	Beijing	33 − 1	S		0.125	Beijing
20 − 2	R		8	Beijing	33 − 2	R		0.5	Beijing
20 − 3	R		8	Beijing	33 − 3	R		0.5	Beijing
21	R	-	0.5	New-1	35	R	-	8	Beijing
21 − 1	R		0.5	New-1	35 − 1	R		8	Beijing
21 − 2	R		0.5	New-1	35 − 2	R		8	Beijing
21 − 3	R		0.5	New-1	35 − 3	R		8	Beijing
22	R	-	1	Beijing	3	R	-	1	Beijing
22 − 1	R		1	Beijing	3 − 1	R		1	Beijing
22 − 2	R		1	Beijing	3 − 2	R		1	Beijing
22 − 3	R		1	Beijing	3–3	R		1	Beijing
23	R	*	4	Beijing	9	R	-	8	Beijing
23 − 1	R		4	Beijing	9 − 1	R		8	Beijing
23 − 2	S		0.0156	Beijing	9 − 2	R		8	Beijing
23 − 3	R		4	Beijing	9 − 3	R		8	Beijing
24	R	*	0.5	Beijing	15	R	-	8	Beijing
24 − 1	S		0.125	Beijing	15 − 1	R		8	Beijing
24 − 2	R		0.5	Beijing	15 − 2	R		8	Beijing
24 − 3	S		0.0156	Beijing	15 − 3	R		8	Beijing
26	R	-	8	Beijing	6	S	-	0.125	Beijing
26 − 1	R		8	Beijing	6 − 1	S		0.125	Beijing
26 − 2	R		8	Beijing	6 − 2	S		0.06	Beijing
26 − 3	R		8	Beijing	6 − 3	S		0.125	Beijing
28	R	*	8	Beijing	8	S	-	0.125	Beijing
28 − 1	R		8	Beijing	8 − 1	S		0.125	Beijing
28 − 2	R		8	Beijing	8 − 2	S		0.06	Beijing
28 − 3	S		0.0156	Beijing	8 − 3	S		0.125	Beijing
30	R	-	8	Beijing	10	R	-	1 1	Beijing Beijing
30 − 1	R		8	Beijing	10 − 1	R			
30 − 2	R		8	Beijing	10 − 2	R		0.25	Beijing
30 − 3	R		8	Beijing	10 − 3	R		0.25	Beijing
31	R	-	2	Beijing	11	R	-	0.5	Beijing
31 − 1	R		2	Beijing	11 − 1	R		1	Beijing
31 − 2	R		2	Beijing	11 − 2	R		1	Beijing
31 − 3	R		2	Beijing	11 − 3	R		0.25	Beijing
32	R	-	4	Beijing	-	-	-	-	-
32 − 1	R		4	Beijing					
32 − 2	R		4	Beijing					
32 − 3	R		4	Beijing					

*:BDQ Heteroresistance

Regarding the MIRU-VNTR analysis, most isolates (94.73%) showed the Beijing genotype, with the exception of PII-21, which had the NEW-1 genotype, as shown in Table 3. Despite the similar genotypes (no mixed infection) observed in most cases, some strains and their separate colonies (1, 2, and 3) displayed a different BDQ resistance pattern (21.05%) (Table 2).

**Table 3:** Results of MIRU-VNTR of the 15 resistance isolates and 4 fully susceptible isolates

MIRU-VNTR loci																										
ID	154	424	577	580	802	960	1644	1955	2059	2163b	2165	2347	2401	2461	2531	2687	2996	3007	3171	3192	3690	4052	4156		4348	Genotype
20	2	4	4	2	3	3	3	5	na	6	4	4	4	2	5	1	7	3	3	5	3	7	2	3		Beijing
21	2	2	4	2	3	2	2	5	2	2	3	4	2	1	5	1	4	3	2	3	3	7	2	2		NEW-1
22	2	4	4	2	3	3	3	5	2	3	4	4	4	2	5	1	7	3	3	5	3	7	2	3		Beijing
23	2	4	4	2	3	3	3	5	2	3	4	4	4	2	5	1	7	3	3	5	3	7	2	3		Beijing
24	2	4	4	2	3	3	3	5	2	6	4	4	4	2	5	1	5	3	3	5	3	8	2	3		Beijing
26	2	4	4	2	3	3	3	5	2	6	4	4	4	2	5	1	7	3	3	5	3	7	2	3		Beijing
28	2	4	4	2	3	3	3	5	na	6	4	4	4	2	5	1	7	3	3	5	3	7	2	3		Beijing
30	2	4	4	2	3	3	3	5	2	3	4	4	4	2	5	1	7	3	3	5	3	7	2	3		Beijing
31	2	4	4	2	3	3	3	5	na	6	4	4	4	2	5	1	5	3	3	5	3	8	2	3		Beijing
32	2	4	4	2	3	3	3	5	2	6	4	4	4	2	5	1	5	3	3	5	3	8	2	3		Beijing
33	2	4	4	2	3	3	3	5	na	3	4	4	4	2	5	1	7	3	3	5	3	7	2	2		Beijing
35	2	4	4	2	3	3	3	5	2	6	4	4	4	2	5	1	5	3	3	5	3	8	2	3		Beijing
3	2	4	4	2	3	2	3	5	2	2	3	4	4	2	5	1	7	3	3	5	3	7	2	2		Beijing
9	2	4	4	2	3	3	3	5	na	6	4	4	4	2	na	1	5	3	3	5	3	8	2	2		Beijing
15	2	4	4	2	3	3	3	5	2	6	4	4	4	2	5	1	7	3	3	5	3	7	na	3		Beijing
6	2	4	4	2	3	3	3	5	2	3	4	4	4	2	5	1	7	3	3	5	3	7	2	3		Beijing
8	2	4	4	2	3	3	3	5	na	6	4	4	4	2	5	1	5	3	3	5	3	8	2	3		Beijing
10	2	2	4	2	3	3	3	5	2	6	4	4	4	2	5	1	7	3	3	3	3	7	2	3		Beijing
11	2	4	4	2	3	3	3	5	2	6	4	4	4	2	5	1	7	3	3	5	3	7	na	3		Beijing

na: not amplified

### Efflux pumps have an important role in BDQ resistance

In this study, all 15 MDR isolates demonstrated resistance to BDQ, with MICs of 0.5 to 8 µg/mL. However, when the combination of BDQ and VP was tested, the MICs ranged from 0.007 to 8 µg/mL. The four sensitive isolates displayed MICs against BDQ ranging from 0.125 to 1 µg/mL. Table 4 presents the data on MICs and the effect of VP treatment. This indicates a significant decrease in MICs against BDQ when combined with VP. Among the 15 MDR/XDR isolates, nine isolates exhibited at least a gene overexpressed (four-fold induction) under BDQ stress. Two isolates (PII-31 and PII-28) among the MDR/XDR isolates showed overexpression of five efflux pump genes under BDQ stress (*p* < 0.05). Additionally, ten isolates under BDQ + VP stress displayed four-fold down-regulation in at least one of the eight evaluated genes (*p* < 0.05). In contrast, among the four fully susceptible isolates, PII-8 and PII-10 did not show induction in any of the eight efflux pump genes under BDQ stress. However, two fully susceptible isolates exhibited overexpression of either the *Rv0676C* or *Rv3065* gene (*p* < 0.05). Furthermore, three isolates showed more than four-fold down-regulation of *Rv0676c* and *Rv1410c* under BDQ + VP stress (*p* < 0.05). Also, in PII-10 and PII-11 isolates showed down-regulation of *Rv1258c*, *Rv1634*, *Rv1819*, *Rv2459* and *Rv2846* under BDQ + VP stress (Table 5).

**Table 4:** MIC results, Drug susceptibility profile, efflux pump expression and VP treatment, in 15 MDR and XDR isolates

No.	BDQ	INH	RIF	STR	EMB	KAN	OFX	CAP	MIC BDQ	MIC BDQ + VP	MIC reduction fold change	Genes overexpressed under BDQ	Genes down regulated under VP + BDQ stress
**PII-20 (MDR)**	R	R	R	R	R	S	S	S	8	0.06	133	*Rv3065*	*
**PII-21 (MDR)**	R	R	R	R	R	S	S	R	0.5	0.5	*	*Rv1410c*	*Rv1410c- Rv1819*
**PII-22 (MDR)**	R	R	R	R	R	S	S	R	1	0.007	142	*Rv0676c*	*Rv0676c - Rv1819- Rv2459- Rv1634*
**PII-23 (MDR)**	R	R	R	R	R	R	S	R	4	0.0625	64	*	*
**PII-24 (MDR)**	R	R	R	R	R	R	S	R	0.5	0.0156	32	*	*Rv1410c*
**PII-26 (XDR)**	R	R	R	S	R	S	R	R	8	8	*	*Rv0676c -Rv1410c*	*Rv1410c - Rv2459*
**PII-28 (MDR)**	R	R	R	S	R	S	S	R	8	0.06	133	*Rv0676c - Rv2846- Rv1634- Rv3065- Rv1819*	*Rv3065- Rv2846*
**PII-30 (XDR)**	R	R	R	R	R	R	R	R	8	0.06	133	*	*
**PII-31 (XDR)**	R	R	R	R	R	S	R	R	2	0.0156	128	*Rv2459- Rv3065- Rv1410c - Rv1634- Rv0676c*	*Rv0676c - Rv1258c - Rv1410c - Rv1819*
**PII-32 (MDR)**	R	R	R	R	R	S	S	S	4	0.125	32	*	*
**PII-33 (XDR)**	R	R	R	R	R	R	R	R	0.5	0.5	*	*Rv0676c - Rv1258c - Rv2846*	*Rv1258c - Rv2846*
**PII-35 (XDR)**	R	R	R	R	R	S	R	S	8	0.06	133		*Rv0676c - Rv1258c- Rv1410c - Rv1634*
**PII-3 (XDR)**	R	R	R	R	R	R	R	R	1	0.125	8	*Rv0676c - Rv2459- Rv1819*	*Rv0676c - Rv1634*
**PII-9 (XDR)**	R	R	R	R	R	R	R	R	8	0.06	133	*	*
**PII-15 (XDR)**	R	R	R	R	R	R	R	R	8	0.125	64	*Rv1258c*	*Rv1258c*

*: not changed

BDQ Bedaquiline, VP Verapamil, INH Isoniazid, CAP Capreomycin, RIF Rifampicin, KAN Kanamycin, EMB Ethambutol, STR Streptomycin, OFX Ofloxacin,, R Resistant, S SusceptibleMIC: Minimum inhibitory concentration

**Table 5:** MIC results, Drug susceptibility profile, efflux pump expression and VP treatment, in 4 fully susceptible isolates

No.	BDQ	INH	RIF	STR	EMB	KAN	OFX	CAP	MIC BDQ	BDQ + VP	MIC fold change	Genes overexpressed under BDQ	Genes down-regulated under VP + BDQ stress
PII-6	S	S	S	S	S	S	S	S	0.125	0.007	17.8	Rv0676c	Rv0676c - Rv1410c
PII-8	S	S	S	S	S	S	S	S	0.125	0.0039	32	*	*
**PII-10**	R	S	S	S	S	S	S	S	1	0.06	16	*	*Rv3065- Rv0676c - Rv1258c - Rv1410c - Rv1634- Rv1819- Rv2459- Rv2846*
**PII-11**	R	S	S	S	S	S	S	S	0.5	0.0625	8	*Rv3065*	*Rv0676c - Rv1258c - Rv1410c - Rv1634- Rv1819- Rv2459- Rv2846*

*: not changed

BDQ Bedaquiline, R Resistant, KAN Kanamycin, VP Verapamil, RIF Rifampicin, CAP Capreomycin, EMB Ethambutol, STR Streptomycin, OFX Ofloxacin, S Susceptible, INH Isoniazid, MIC: Minimum inhibitory concentration

These findings shed light on the complex interactions between BDQ resistance and efflux pump and may have implications for understanding drug resistance mechanisms and potential treatment strategies.

## Discussion

Gaining a comprehensive understanding of drug resistance mechanisms in *M. tuberculosis* is crucial to improving treatment efficacy and shorten treatment duration [20]. Previous studies have reported the development of MDR and specific heteroresistance in clinical isolates [22]. Heteroresistance is considered the initial step towards transitioning from a susceptible state to MDR, and all forms of resistance contribute to treatment failure. In the current study, out of the 15 resistant isolates and fully susceptible isolates, 21.05% (4/19) exhibited BDQ heteroresistance patterns. In a study by Nimmo et al., which investigated the diversity of drug resistance in *M. tuberculosis* during treatment, six out of ten isolates displayed a heteroresistance pattern to BDQ based on genotype analysis [23]. The heteroresistance rate of 21.05% observed in this study aligns closely with findings reported by Faye et al. [24]. Furthermore, Kargarpour et al. reported a heteroresistance rate of 45.71% among 35 patients with mixed infections [5]. These findings highlight the prevalence of heteroresistance and its significance in the context of MDR/XDR-TB treatment. Understanding the underlying mechanisms and factors contributing to heteroresistance is crucial for enhancing treatment strategies and combating drug-resistant tuberculosis effectively.

In our study, all of the heteroresistant isolates belonged to the Beijing genotype, which is also-known as lineage 2 of *M. tuberculosis*. The Beijing family is one of the most common genotypes worldwide and is associated with immune evasion and antibiotic resistance, contributing to rapid bacterial growth, spread, and transmission. This family of *M. tuberculosis* is considered more contagious than other lineages [25]. In our study, despite having the same genotypes in most cases, different patterns of antibiotic resistance were observed in some strains and individual colonies. The discovery of heteroresistance in isolates without mixed infections suggests that drug resistance mechanisms are not solely reliant on the presence of different strains but can also arise through other genetic or phenotypic changes within a single strain. Understanding the factors contributing to heteroresistance, regardless of mixed infections, can provide valuable insights into the emergence, spread, and management of drug-resistant TB. This study investigated the absence or presence of mutation in the *atpE* and *Rv0678* genes, critical genes associated with BDQ resistance. Interestingly, all clinically reported mutations were found within the *Rv0678* gene [26]. None of the studied isolates in our research harbored the mutations in the *atpE* and *Rv0678* genes. Although drug resistance is primarily attributed to mutations causing modifications in drug targets, it has become evident that multidrug efflux systems can also play a significant role in *M. tuberculosis* [27]. At the phenotypic level, our study evaluated the impact of the efflux pump inhibitor, VP, on the reduction of BDQ MIC in MDR and XDR isolates. Encouragingly, VP had a notable effect in reducing BDQ resistance in 80% of these isolates, a finding consistent with previous study [10]. At the RNA level, we analyzed the expression levels of eight genes that encode potential drug efflux transporters under VP efflux inhibition and drug stress. *Rv0676c* showed the highest expression level among the 15 MDR and XDR isolates, with overexpression detected in 40% of these isolates under BDQ stress (*p* < 0.05). However, VP-treated MDR and XDR isolates exhibited significant down-regulation of the *Rv0676c* efflux gene, indicating the role of VP in suppressing efflux pump activity (*p* < 0.05). In fully susceptible isolates, *Rv0676c* and *Rv1410c* demonstrated the most substantial down-regulation than other genes, suggesting their involvement in drug resistance. This aligns with the findings of Song et al., who showed a potent role of *Rv0676c* in drug resistance, particularly against BDQ [28].

High-level expression of the gene in the MFS (*Rv1410c*), occurred in three of the MDR and XDR isolates under BDQ stress. Additionally, the efflux pump *Rv1410c* was down-regulated under VP + BDQ stress in five isolates. Also, we showed that none of the fully susceptible isolates overexpressed *Rv1410c*; while this efflux pump showed down-regulation due to by 3/4 VP-treated fully susceptible isolates (*p* < 0.05). Unlike our results, Laws et al. declared that *Rv1410c* was overexpressed in the MDR isolates due to INH and RIF stress [10]. In the presence of VP, PII10 and PII11 isolates reduced the MIC of BDQ which showed the down-regulated of efflux genes *Rv1258c, Rv1634, Rv1819, Rv2846*, and *Rv2459* in response to VP-treated isolates. In a similar study, Li et al. showed higher expression levels of some efflux pump genes in MDR isolates compared to fully susceptible isolates and that differences in expression could be useful in the diagnosis and treatment of drug-resistant TB [29]. The PII33 and PII15 isolates showed the up-regulation of the efflux gene *Rv1258c* and down-regulation following VP treatment (*p* < 0.05). In a similar study, Ramón-García et al. declared the significant effect of the *Rv1258c* efflux pump on aminoglycosides, tetracycline, rifampicin and clofazimine in *M. tuberculosis* [30]. The PII-22, PII-3, and PII-35 isolates exhibited down-regulation of the efflux gene *Rv1634* in the presence of VP (*p* < 0.05). Similarly, Viveiros et al. showed that the resistant phenotype was by VP compound known to inhibit efflux pump [31]. Another MFS family efflux transporter, *EfpA* (*Rv2846c*), was overexpressed in XDR and MDR isolates (PII-28 and PII-33) under BDQ stress and down-regulation of this gene in the presence of VP (*p* < 0.05). Similar to our findings, Li et al. reported that *EfpA* was overexpressed in the MDR isolates due to INH and RIF stress [29]. *Rv2459* (*jef A*), another MFS family efflux transporter, was down-regulated in XDR and MDR isolates (PII-22 and PII-26) under BDQ stress. Rahul et al. reported the increased expression of *jefA* (*Rv2459*) leading to increased resistance against EMB and INH in *M. tuberculosis* [27]. *Rv1819c* is one of the members of ABC transporters whose activity can be associated with tuberculosis treatment failure [32]. The PII-21, PII-22, and PII-31 isolates showed down-regulation of the efflux gene *Rv1819c* in the presence of VP (*p* < 0.05). Gupta et al. revealed that VP supplementation significantly reduced the MIC of BDQ in drug-susceptible and drug-resistant clinical isolates. VP decreased the MIC of BDQ by 8- to 16-fold for all isolates tested [33]. We found that the Mmr efflux transporter (*Rv3065*) was overexpressed in three MDR and XDR isolates (i.e., PII-28, PII-20, and PII-31) in response to BDQ (*p* < 0.05). The BDQ MIC in the PII-28 isolate was decreased by 133-fold using VP; this efflux pump was among the genes down-regulated after receiving VP.

## Conclusions

Our findings clearly demonstrated the crucial role of the efflux pump in BDQ resistance. Additionally, a direct link was established between the *Rv0676c* gene and BDQ resistance (*p* < 0.05). The inclusion of VP significantly reduced the MIC of BDQ in both drug-susceptible and drug-resistant clinical isolates. However, despite VP being approved by the FDA as an EPI, its potential as an anti-TB drug requires more investigation. Interestingly, this study also revealed varying BDQ resistances (heteroresistance) within the same genotypes, necessitating the use of molecular techniques like whole genome sequencing for more comprehensive analysis. Failure to detect such strains can severely impact the treatment of TB patients.

## Data Availability

Data supporting this paper are included in the article.

## Abbreviations

EMB: Ethambutol
ABC: ATP-binding cassette
ADC: Albumin-dextrose-catalase
BDQ: Bedaquiline
CTAB: Cetyltrimethylammonium bromide
CAP: Capreomycin
DST: Drug susceptibility testing
MDR: Multidrug-resistant
INH: Isoniazid
KAN: Kanamycin
MABA: Microplate alamar blue assay
MIRU-VNTR: Mycobacterial Interspersed Repetitive Units- Variable Numbers of Tandem Repeats
OFX: Ofloxacin
MFS: Major facilitator superfamily
MATE: Multidrug and toxic compound extrusion
MIC: Minimum inhibitory concentration
PACE: Proteobacterial antimicrobial compound efflux
RND: Resistance-nodulation-division
RIF: Rifampicin
SMR: Small multidrug resistance
VP: Verapamil
XDR: Extensively drug-resistant
STR: Streptomycin
FDA: Food and Drug Administration
